# The effect of low level laser on number of *Candida albicans* colonies in-vitro: a new finding

**DOI:** 10.1186/s12903-019-0814-5

**Published:** 2019-06-13

**Authors:** Shamsoulmolouk Najafi, Nafiseh Sheykhbahaei, Mina khayamzadeh, Narges Gholizadeh

**Affiliations:** 1 Dental research center, Tehran University of Medical Science, Tehran, Iran; 2 Oral & Maxillofacial Medicine, School of Dentistry, Tehran University of Medical Science, Tehran, Iran

**Keywords:** Low-Level laser therapy, Nystatin, *Candida albicans*

## Abstract

**Background:**

*Candida albicans* is a commensal organism that causes a wide variety of diseases in humans. One of these diseases is oral candidiasis, which occurs at a high recurrence rate in spite of available treatments. The purpose of this study was to compare the effect of low-level laser therapy (LLLT) with the use of nystatin on in-vitro growth of *Candida albicans*.

**Method:**

We prepared two samples of *Candida albicans* at different concentrations: 10^4^ viable cells/ml and 10^6^ viable cells/ml. Specimens from each sample were divided into a control group, a nystatin-treated group, and a group treated with LLLT. The control group was cultured without any intervention. The second group was treated with nystatin and the solution was vibrated for 30 s or 60 s. The third group was irradiated with a gallium-aluminum-argon (Ga-Al-Ar) diode laser (Epic 10; Biolase Inc.)in continuous mode using a wavelength of 940 nm and a power of 1 W for 30 s or 60 s (38 J/cm^2^ and 76 J/cm^2^). The specimens from the nystatin group and the LLLT group were cultured and the number of colony-forming units (CFU/ml) for each group was counted and compared.

**Results:**

Nystatin completely eliminated the colonies (0 colonies) in all specimens. There was an increase in the number of colonies in the LLLT group for both cell concentrations at 30 s and at 60 s. However, this increase was statistically significant only for a concentration of 10^4^ viable cells/ml at an exposure time of 30s. The increase in the concentration of 10^6^ viable cells/ml at both 30 s and 60 s was statistically significant compared with the control group, although the highest number of colonies remained after an exposure time of 60s.

**Conclusion:**

LLLT led to an increase in the growth of *Candida* colonies. However, there was no significant difference related to the exposure time between the different cell concentrations.

## Background

There are different species of *Candida* that are known to cause infection in the oral cavity of humans. *Candida albicans*(*C. albicans)* is the most common pathogen associated with oral candidiasis [1]. Several conditions increase a person’srisk of oral candidiasis, including senescence, immunosuppression, organ transplantation, diabetes mellitus, long-term use of corticosteroids, the use of broad-spectrum antibiotics, and wearing dentures [2]. The high incidence rate of oral candidiasis is related to the ability of *C. albicans* to adapt to various environments in the host’s oral cavity [3]. Candidiasis and morbidity associated with candidiasis have become more prevalent as the population ages and the numbers of people who have compromised immune systems increase [1–8].

*Candida* can cause denture stomatitis, a chronic inflammatory condition that occurs especially in the palatal mucosa of people who wearill-fitting dentures [9]. This condition occurs in approximately one third to two thirds of people who wear full dentures [10]. It is believed that the prevalence of denture stomatitis is higher in women than in men [11]. The treatment of denture stomatitis is rather complicated because of the multifactorial nature of the disease. Conventional treatments include improving oral hygiene, using antiseptic mouthwash, removing the dentures at night and placing them in a disinfectant solution, and replacing ill-fitted dentures. Nystatin is an antifungal agent that commonly used to treat denture stomatitis [12].

Nystatin is from the Polyenes family that acts by binding to ergosterol, which is one of the major constituents of the fungal cell wall. It can also bind to cholesterol in the cytoplasmic membrane of the host cells. Unfortunately, the use of nystatin is associated with side effects, including diarrhea, abdominal pain, tachycardia, bronchospasm, facial swelling, muscle pain, Stevens–Johnson syndrome, itching, burning sensations, and rashes [13]. The long-term use of nystatin not only requires the patients cooperation, but it can also lead to the development of drug-resistant strains of *C. albicans* [14]. In addition, the drug is relatively expensive [15, 16]. It is believed that the diluting effect of saliva affects the bioavailability of oral antifungal agents, which may cause therapy failure or the recurrence of infection [5].

Current research is being done on other treatment strategies for oral candidiasis. For example, laser therapy is used to change tissue metabolism, which accelerates wound healing, eradicates infectious organisms, and decreases pain and inflammation [17–19]. Low-level laser therapy (LLLT) uses a gallium-aluminum-argon (Ga-Al-Ar) diode laser with a short 10-μs pulse to control the amount of energy that reaches the exposed tissues. LLLT is also well tolerated by the patient, causes less damage to the surrounding tissues, and provides better healing of the lesions [20].

In this study, a high-power Ga-Al-Ar diode laser with a wavelength of 940 nm at 1 W was used. The aim of the study was to evaluate the effects of the laser using a safe wavelength of 940 nm against colonies of *C. albicans*.

## Methods

A standard strain of *C. albicans*, ATCC18804 [21], was purchased from the Microbiology Research Center, Pasteur Institute of Iran, Tehran, Iran. To prepare the specific concentrations of the strain, we first diluted the samples using sterile saline (0.85%NaCl) to achieve a concentration of 1.5 × 10^8^ viable cells/ml. We then adjusted the concentration to 0.5 McFarland turbidity standard solution and cultured the samples on Sabouraud dextrose agar. Finally, we reserved and incubated all the prepared samples at 37 °C for 24 h. At the end of this period, two different concentrations of *Candida* colonies were obtained. Sample A contained 10^4^ viable cells/ml, while sample B contained 10^6^ viable cells/ml. A total of 72 specimens, 36 from sample A and 36 from sample B, were prepared. Twelve specimens from eachsample were then randomly classified into one of the three subgroups shown in Table 1. Group 1 was the control group. Group 2 was treated with 100,000 units/ml of nystatin. Group 3 was treated with LLLT at a wavelength of 940 nm. All the samples were coded before the appropriate interventions.

**Table 1:** Number of candida colonies in different initial concentration

Base		N	Min	Max	Mean	SD
10^4^	control	12	220.00	720.00	403.3333	172.24014
	Nystatin 30s	6	NA	NA	NA	NA
	Nystatin60s	6	NA	NA	NA	NA
	LLLT 30s	6	520.00	1240.00	946.6667	286.12352
	LLLT 60 s	6	380.00	880.00	636.6667	203.73185
10^6^	Control	12	2320.00	31,120.00	15,700.00	11,165.13860
	Nystatin 30 s	6	NA	NA	NA	NA
	Nystatin 60s	6	NA	NA	NA	NA
	LLLT 30s	6	20,100.0	48,160.00	33,816.6667	9649.97340
	LLLT 60 s	6	31,060.0	45,280.00	38,056.6667	5887.21213

*N* number of specimens, *SD* Standard Deviation, *NA* Not Available

### Control group

The control group consisted of 12 specimens from sample A and 12 from sample B; thus, there were 24 specimens in total. The specimens were cultured to ensure that the *C. albicans* were able to grow in vitro (Table 1).

### Nystatin group

The nystatin group consisted of 10 ml nystatin suspension added to 12 specimens from sample A and 12 specimens from sample B. The solutions were immediately mixed in a vibrator; six specimens from each sample were vibrated for 30s and six specimens from each sample were vibrated for 60s (Table 1). The specimens were then cultured in Sabouraud dextrose agar. After incubating the cultures for 48 h at 37 °C, the number of colonies of *C. albicans* per plate was measured using the colony-forming units (CFU/ml). The two investigators who counted the number of colonies were blinded to the intervention.

### LLLTgroup

AGa-Al-Ardiode laser (Epic 10; Biolase Inc.) with a wavelength of 940 nm at1W was used in this study. The laser was pre-configured and required no calibration. The diagonal head probe was 1 cm in diameter. Twelve specimens each from sample A and sample B were exposed to the laser irradiation. Six specimens from each sample were exposed for 30s(38 J/cm^2^), while six from each sample were exposed for 60s (76 J/cm^2^) (Table 1). Similar to the nystatin group, all specimens were cultured, encoded, and incubated. The number of colonies was determined by two investigators blinded to the intervention.

Once the specimens had been decoded, the data were analyzed using SPSS program, version 20. Because the data from this study were nonparametric, we applied the nonparametric tests (NPAR test) and the Kruskal–Wallis test to compare the variables. We also used repeated measures ANOVA (Analysis of variance) and the post hoc test to compare the data obtained from the different concentrations of *Candida* colonies, type of intervention, and time of exposure.

## Results

In this study, 36 specimens from sample A (10^4^ viable cells/ml) and 36 specimens from sample B (10^6^ viable cells/ml) were each divided into three groups: a control group, a nystatin-treated group, and aLLLT group. A total of 72 specimens was used; each group contained 12 specimens. All groups were studied at 30s and 60s.

In the nystatin group, the number of colonies from sample A and sample B significantly decreased at 30s and 60s compared with the control and LLLT groups. The nystatin completely eliminated the colonies (0 colonies), regardless of the concentration or vibration period (time of exposure).

In the LLLT group, the number of colonies from sample A and sample B increased at 30s and 60s. However, this increase was only statistically significant compared with the sample A and control group at an exposure time of 30s. The increase in sample B at both 30 s and 60 s was statistically significant compared with the control group, although the highest number of colonies was found after the 60 s exposure to the laser (Tables 2 and 3).

**Table 2:** Effect of different time on number of colonies at concentration of 10^4^

Groups comparison	Mean difference (I-J)	SD error	*P*value	95% IC	
				Lower bound	Upper bound
Laser 30s to Control	543.33333	136.34108	.011^*^	136.2113	950.4553
Laser 60s to control	233.33333	108.91383	.166	79.8548	546.5215
Laser 30s to Laser 60s	310.00000	143.39534	166	−108.7890	728.7890

*: (*P* value≤0.05)

Applied tests in these tables: Repeated measured ANOVA, POST HOC

**Table 3:** Effect of different time on number of colonies at concentration of 10^6^

Groups comparison	Mean difference (I-J)	SD error	*P*value	95% IC	
				Lower bound	Upper bound
Laser 30s to Control	18,116.66667	6024.70340	.040*	814.8320	35,418.5013
Laser 60s to control	22,356.66667	5152.98597	.008^*^	6657.0263	38,056.3070
Laser 30s to Laser 60s	− 4240.00000	4614.85380	.767	−17,993.5245	9513.5245

*: (*P* value≤0.05)

Applied tests in these tables: Repeated measured ANOVA, POST HOC

## Discussion

Few investigations have evaluated the effect of LLLT on the behavior of colonies of *C. albicans*. Based on a review of the literature, we did not find any studies that hadsimilar results to our study on *C. albicans*. We did find two studies that reported that LLLT caused an increase in bacterial proliferation [22] and an increase in squamous carcinoma cell lines [23]. Nussbaum et al. (2002) showed that bacterial proliferation significantly increased after LLLT using a 3-W diode laser at a wavelength of 810 nm, the laser was used in continuous mode at an intensity of 1 J/cm^2^ [22]. Henriques et al. (2014) investigated the effects of LLLT on the expression of several tumor markers including β-catenin, MMP-9, cyclin D1 and E-cadherin, in a squamous carcinoma cell line [23]. They applied diode laser at a wavelength of 660 nm and energy densities of 0.5 J/cm^2^ and 1.0 J/cm^2^. The researchers found that alterations in the expression of tumor markers by LLLT had a stimulatory effect on the proliferation and invasion of SCC cells [23]. Generally, the results of these two studies were consistent with our results, which showed that LLLT caused an increase in the growth of the organism by unknown mechanisms.

These results obtained in this study ofthe LLLTon *C. albicans* may be related to such variables as levels and doses of energy, power, and power density, as well as the wavelength, method of application of laser (contact or non-contact and pulsed or continuous modes), contamination with other microorganisms, and time of the exposure. For example, red light is more effective than an infrared laser on pro-inflammatory effects or the growth of organisms because of the greater absorption of red light at a shorter wavelength by the photoreceptor (cytochrome C oxidase). A reason for the results of this study could be that the laser caused the acceleration of the electron transport chain in the mitochondria, which led to a higher rate of Adenosine triphosphate (ATP) production [18]. In addition, the exact mechanism for the differences seen between pulsed and continuous mode of LLLT has not yet been determined. In pulsed mode, the tissues are affected by a series of pulses in which the thermal energy increases with each period of radiation (pulse) because of the thermal energy remaining from the previous pulse. Thus, in pulsed mode, there is a higher level of heat inside the tissue than during continuous mode, which increases the damage to the tissue. In continuous mode, the dissemination of thermal energy prevent the accumulation of excess heat in the tissue [22]. Another reason for the results in this study could relate to the use of a high-power diode laser(> 0.5 W). High-power laser therapy delivers high energy to the tissue and causes photochemical effects through the stimulation oxidation of mitochondria and ATP production [24]. Thus,we can claim that our different methodology, which involved the continuous mode of a high-power diode laser, could lead to an increase inATP production in *C. albicans*, which caused less fungal cells to be destroyed.

The results from most of the studies that evaluated the effect of LLLT on *C. albicans* did not agree with the findings of our study. For example, Maver-Biscaninetal.(2004) compared the effect of laser irradiation at two different wavelengths, 685 nm (30 mW) and 830 nm (60 mW),with asham laser and an antimicotic agent on 70 patients who had evidence of denture stomatitis [25]. They found that a similar fungicidal effect occurred in both the laser and antimicotic-treated groups, and that the effect was statistically significant in comparison with the placebo group (*P* < 0.001). This study was the first to research the effect of LLLT on *C. albicans* without using a photosensitizer [25].

Similarly, Mousaviet al.(2014) Exposed *C. albicans* cultures to LLLTatenergy levels of 3 J, 5 J, 10 J, and 20 J at a wavelength of 685 nm (50 mW) and at intensities of 3 J, 5 J, 10 J, 30 J, and 50 J at a wavelength of 830 nm (400 mW). The researchers concluded that a laser at an energy level higher than 10 J at wavelengths of 685 nm and 830 nm could significantly reduce the growth of *C. albicans* colonies [26].

Another researcher evaluated the effect of a 1-W laser and a 0.75-W laser with and without NaOCl on *C. albicans* colonies that had been incubated in single-rooted teeth [27]. The results showed that a power of 1 W combined with NaOCl caused the greatest reduction in the number of colonies, whereas NaOCl alone resulted in a minimum reduction in CFUs of *C. albicans* [27]. In 2010, a study showed that LLLT had a similar fungicidal effect on *C. albicans* compared with photodynamic therapy using toluidine blue, methylene blue, and malachite green [28]. The researchers applied a gallium-aluminum-arsenide (Ga-Al-As) laser at a wavelength of 660 nm with different energy densities of 15.8 J/cm^2^, 26.3 J/cm^2^, and 39.5 J/cm^2^ [28].

Maver-Biscanin et al.(2005) discussed the effect of LLLT in non-contact mode on 2 patients with denture stomatitis for different exposure times:5 min (830 nm, 3.0 J/cm^2^, and 60 mW) and 10 min (685 nm, 3.0 J/cm^2^, and 30 mW). The researchers showed that LLLT was effective in treating denture stomatitis and reduced the number of colonies and the palatal inflammation [29].

Bassoet al. (2011 investigated the effect of LLLT on biofilms with colonies of *Streptococcus mutans* and/or *C.albicans*. Single-species biofilm (SSB) and dual-speciesbiofilm (DSB) were exposed to an indium-gallium-arsenide-phosphide (In-Ga-As-P) diode laser with energy density of 5 J/cm^2^, 10 J/cm^2^, or 20 J/cm^2^.(780 nm ± 3 nm and 0.04 W). The study showed that LLLT reduced the bacterial and fungal viability, as well as the growth of the SSB. However, *S. mutans* was resistant to the LLLT when associated with *C. albicans* (DSB), although when *C. albicans* was associated with *S. mutans* (DSB), there was a significant decrease in biofilm growth. While the LLLT did not affect the morphology of the microorganisms in the SSB group, the formation of *C. albicans* hyphae was reduced in the DSB. It is believed that the interaction between different microbial species may alter the inhibitory effect of LLLT [30]. Accordingly, it is important to consider that the contamination of *C. albicans* with other microbial species can lead to morphological changes in the fungi, which can affect the results from LLLT.

Carneiro et al. (2016) also studied the effect of LLLT on *C.albicans* [17]. They used an indium-gallium-aluminum-phosphide (In-Ga-Al-P) laser at a wavelength of 685 nm and a power of 30 mW and a Ga-Al-As laser at a wavelength of 830 nm and a power of 40 mW at energy doses of 6 J/cm^2^, 8 J/cm^2^, 10 J/cm^2^,and 12 J/cm^2^. There was no statistically significant reduction observed in the number of colonies of *C. albicans*; the best result was obtained at6J/cm^2^ using the Ga-Al-As laser [17].

A study done in 2016 claimed that the stimulation of polymorphonuclear leukocytes (PMNs) by LLLT led to the production of higher levels of reactive oxygen species (ROS),such ashypochlorite anions (ClO^−^) and hydroxyl radicals (HO•), which enhanced the fungicidal capacity of the treatment against *C. albicans*. PMNs were treated witha laser at a wavelength of 660 nm or 780 nm at40 mW andincreasing energy levels up to 19.2 J [18].

In this study, we compared the effect of LLLTwithnystatin treatment on the growth of different concentrations (10^4^ viable cells/mland10^6^viable cells/ml) of *C. albicans*; the treatments were given for 30 s and 60 s. While the nystatin eliminated The *C. albicans* colonies for both concentrations and time periods, surprisingly the LLLT led to an increase in the number of colonies. To the best of our knowledge, this result had never been reported before. According to the results of previous studies, several factors are involved in the effectiveness of LLLT on the growth of *C. albicans* colonies, including modulation frequency, radiant exposure, and other factors such as power, energy level and energy densities [31]. Interestingly, LLLT has reported to have both anti-inflammatory and pro-inflammatory effects. The anti-inflammatory effects of LLLT involve the diminished chemotaxis of PMNs, which affects the site of inflammation. However, somewhat paradoxically, LLLT may also be able to enhance the antimicrobial activity levels of PMNs. These effects were discussed in studies undertaken in 2015 and 2016 [18, 32]. The researchers suggested that this mechanism could be one of the reasons for the reduced growth of *Candida* under LLLT. Furthermore they noted that the antifungal mechanism of LLLT might be related to the thermal effect of photo disruption. It is believed that LLLT destroys the cell wall of a microorganism and causes denatured proteins to accumulate in the cytoplasm. The damage to the microorganism results in cell death. In this situation, the microorganism attempts to maintain homeostasis despite the increasing cellular stress, which subsequently inhibits growth and leads to cell lysis. An alteration in the relationship between microorganism and its cellular substrate may also cause a disruption of the vital activities of microorganisms [30].

In addition to its anti-inflammatory effects, LLLT has pro-inflammatory effects. LLLT promotes wound healing by accelerating the synthesis of collagen and the wound healing time and obtaining tensile strength. These findings indicated that there was an increase in metabolic indices, such as ATP synthesis, fibroblast proliferation, collagen synthesis, and other biomechanical indices of tissues [31].

## Conclusion

Several factors, including the type of laser therapy, cultural conditions, and the type of organism, have significant effects on the results of LLLT on infectious agents. The paradox of LLLT being both a potent anti-inflammatory and a pro-inflammatory agent, as well as the benefits and risks involved in using lasers on infected tissues, must be considered before LLLT can be recommended for use in clinical practice. We believe that further research is required to evaluate the behavior of *C. albicans* and its responses to LLLT under controlled conditions.

## Data Availability

Not applicable.

## Abbreviations

ANOVA: Analysis of variance
ATP: Adenosine triphosphate
*C. albicans*: *Candida albicans*
CFU: Colony-forming units
ClO^−^: Hypochlorite anions
DSB: Dual-species biofilm
Ga-Al-Ar: Gallium-aluminum-argon
HO•: Hydroxyl radicals
LLLT: Low-level laser therapy
NaOCl: Sodium hypochlorite
NPar: Nonparametric
PMNs: Polymorphonuclears
ROS: Reactive oxygen species
SCC: Squamus cell carcinoma
SSB: Single-species biofilm
